# Cardioprotective effects of sodium thiosulfate against doxorubicin-induced cardiotoxicity in male rats

**DOI:** 10.1186/s40360-022-00569-3

**Published:** 2022-05-25

**Authors:** Maryam Shekari, Narges Khalilian Gortany, Mina Khalilzadeh, Alireza Abdollahi, Homanaz Ghafari, Ahmad Reza Dehpour, Mahmoud Ghazi-Khansari

**Affiliations:** 1 Department of Pharmacology, School of Medicine, Tehran University of Medical Sciences, Tehran, Iran; 2 Experimental Medicine Research Center, Tehran University of Medical Sciences, Tehran, Iran; 3 Department of Pathology, School of medicine, IKHC, Teheran University of Medical Sciences, Tehran, Iran

**Keywords:** Doxorubicin, Sodium thiosulfate, Cardiotoxicity, Reactive oxygen species, Oxidative stress

## Abstract

**Background:**

Doxorubicin (DOX) is an effective antitumor agent, but its clinical usage is limited due to adverse cardiotoxic effects. Several compounds have been studied to reduce DOX cardiotoxicity to improve its therapeutic index. This study was aimed to investigate the protective effects of sodium thiosulfate (STS) pre-treatment against DOX-induced cardiomyopathy in rats.

**Methods:**

Male Wistar rats were randomized into 4 groups: control (saline), DOX (2.5 mg/kg, 3 times per week, intraperitoneal [i.p.]), STS (300 mg/kg, 3 times per week, i.p), and DOX + STS (30 min prior to DOX injection, 3 times per week, i.p.) over a period of 2 weeks. The body weight, electrocardiography, histopathology, papillary muscle contractility, and oxidative stress biomarkers in heart tissues were assessed.

**Results:**

The results indicated that STS significantly improved the body weight (*P* < 0.01), decreased QRS complex and QT interval on ECG (*P* < 0.05 and *P* < 0.001, respectively), as well as declined the papillary muscle excitation, and increased its contraction (*P* < 0.01) compared to DOX-treated rats. STS strongly suppressed oxidative stress induced by DOX through the significant improvement of the cardiac tissue antioxidant capacity by increasing glutathione, superoxide dismutase (*P* < 0.001), and decreasing the level of lipid peroxidation (*P* < 0.01).

**Conclusion:**

Taken together, the results of this study demonstrated that STS showed potent cardioprotective effects against DOX-induced cardiotoxicity by suppressing oxidative stress.

**Supplementary Information:**

The online version contains supplementary material available at 10.1186/s40360-022-00569-3.

## Background

Doxorubicin (DOX) is an anthracycline antibiotic with potent antitumor activity that extensively used to treat wide range of solid tumors such as breast, thyroid, and ovarian cancers as well as human hematological malignancies [1]. However, the clinical use of DOX is limited due to the cumulative dose-related cardiotoxicity as a serious side effect, which results in arrhythmia, irreversible cardiomyopathy, and congestive heart failure (CHF) [1, 2]. The exact pathogenesis of Dox-induced cardiotoxicity has not been fully defined but could be due to multiple mechanisms. Generation of reactive oxygen species (ROS) has been suggested as one of several contributing factors which also include production of iron regulatory protein (IRP), release of nitric oxide (NO), mitochondrial dysfunction, decrease adenosine triphosphate (ATP) production, calcium overload, inflammatory mediators, and inhibition of topoisomerase-IIβ in cardiomyocytes [2–5]. Due to lack of adequate antioxidant mechanisms in myocardial tissues, the accumulation of DOX in mitochondria of cardiomyocytes results in redox cycling of DOX to excessive ROS generation leads to dysfunction of mitochondrial cardiomyocytes [5, 6]. The potential ROS toxicity is characterized by reducing the activity of the antioxidant enzymes such as peroxidase, catalase and superoxide dismutase (SOD) [7].

Sodium thiosulfate (Na_2_S_2_O_3_, STS) is a non-toxic compound and FDA approved chelating agent for the traditional treatment of cyanide poisoning, as well as an anti-calcification agent to treat calciphylaxis [8, 9]. Moreover, STS was successfully administered to treat toxic overdoses of cisplatin, a chemotherapy drug, in the clinical setting [10]. Thiosulfate is the metabolite of endogenous hydrogen sulfide (H_2_S) in mitochondria and has been known as an antioxidant agent in scavenging and reducing ROS, as well as activating the gene expression of antioxidant enzymes [11]. In addition, STS is reported to have the vasodilatory, antiapoptotic, and anti-inflammatory effects [12–14]. The cardioprotection of STS against cardiac ischemia-reperfusion (IR) injury and CHF has been shown previously through preserving the mitochondrial activity, interacting with caspase enzymes, scavenging free radical, and chelating calcium ion, as well as increasing the ventricular H_2_S generation [15–17].

The goal of this study is to evaluate the cardioprotective effects of STS against DOX-induced cardiotoxicity in male rats. Recently, Mizuta et al. studied the protective effect of STS against DNA damage and apoptosis induced by DOX in cardiomyocytes of mice and observed the cardioprotective of STS in a short-time period investigation of 6 days after the DOX/STS treatment [18]. In this study, the therapeutic effects of STS against DOX-induced cardiotoxicity were studied in a longer-time period (14 days) in rats by evaluation of antioxidant markers, electrocardiography (ECG), papillary muscle contraction and excitation, and histological changes.

## Methods

### Drugs and chemicals

STS and DOX HCl were purchased from Sigma (St. Louis, MO, USA). All the chemicals used in the preparation of the physiologic salt solution were purchased from Merck (Darmstadt, Germany) (NaCl, MgCl_2_, KCl, NaH_2_PO_4_, NaHCO_3_, glucose, CaCl_2_, KH_2_PO_4_, and EDTA).

### Animals and experimental design

Male Wistar rats, weighing 200–250 g, obtained from the department of pharmacology of Tehran University of Medical Sciences (TUMS) were used for this study. Animals were housed in standard polycarbonate cages under controlled conditions of ambient temperature (22 ± 2 °C), a 12-h-dark/12-h-light cycle, and free access to food and water ad lib. All the experiments were performed between 9:00 and 14:00. All animal experiments were performed with the relevant guidelines and regulations and compliance with ARRIVE guidelines for the care and use of experimental animals by the committee for supervision of Experiment on animals (CPCSEA) and the National Institute of Health NIH Protocol. Ethical approval was obtained from the Research and Ethics Committee of Tehran University of Medical Sciences for care and use of laboratory animals (approval number IR.TUMS.MEDICINE.REC.1397.178).

**Fig. 1:**
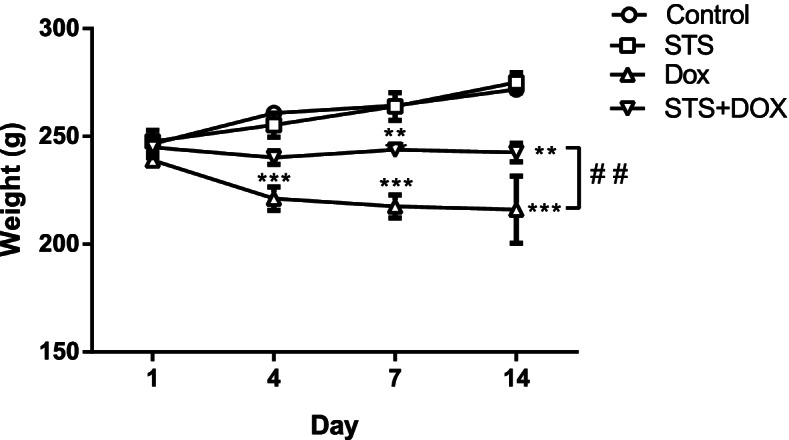
Experimental design and timeline. Rats were allocated into control, DOX, STS, and STS + DOX groups. Normal saline (control), 2.5 mg/kg DOX (DOX) and 300 mg/kg STS (STS and STS + DOX groups) were i.p. injected. Thirty minutes later, 2.5 mg/kg DOX was i.p. injected to rats in STS + DOX group. ECG was recorded for all groups before the first injection and after the last injection. Thereafter, all four groups of rats were euthanized, and their heart tissues were collected for H&E staining, oxidative stress markers analysis, and left ventricular papillary muscle contractile assessment

Rats were randomly allocated to 4 groups, each group containing 8 rats as below (Fig. 1):

- Group 1 received normal saline three times per week for 2 weeks (control group).
- Group 2 received DOX (2.5 mg/kg, i.p.) three times per week for 2 weeks (DOX group).
- Group 3 received STS (300 mg/kg, i.p.) three times per week for 2 weeks (STS group).
- Group 4 received STS (300 mg/kg, i.p.) 30 min prior to DOX injection, three times per week for 2 weeks (STS + DOX group).

Total cumulative dose of DOX (15 mg/kg) in a short time (2 weeks) has been shown to be sufficient to induce the acute cardiac damage [19]. STS dose (300 mg/kg) was determined via a preliminary dose-response study in which revealed the cardioprotective effect of this dose (supplementary Fig. 1). Animals were weighed on days 1, 4, 7 and 14. General conditions and mortality rate of the animals were recorded daily for 2 weeks. After 2 weeks and on the day of sacrifice, ECG was recorded in all treated groups. Then, rats were anesthetized by diazepam (2.5 mg/kg i.p., Sigma, USA) and ketamine (50 mg/kg i.p., Sigma, USA) and heart tissues were collected for left ventricular papillary muscle contractile assessment, oxidative stress markers analysis, and histopathological studies.

### ECG test

ECG was recorded before the first injection and after the last injection in all four groups by using a PowerLab data acquisition system (Chart 7.2, ADInstruments, PowerLab). Rats were anesthetized by diazepam and ketamine. Needle electrodes were inserted under the skin for the limb lead at position II. QRS complexes and QT intervals were recorded for the analysis.

### Left ventricular papillary muscle contractile assessment

Rats were anesthetized and hearts were dissected carefully to isolate the left ventricular papillary muscles to the physiological salt solution of NaCl: 112 mM, MgCl_2_: 1 mM, KCl: 5 mM, NaH_2_PO_4_: 0.5 mM, NaHCO_3_: 25 mM, glucose: 10 mM, CaCl_2_: 1.8 mM (pH: 7.4), KH_2_PO_4_: 0.5 mM and EDTA: 0.004 mM which carbogenated with 5% CO_2_ and 95% O_2_. Then the papillary muscles were attached vertically to an isometric force transducer (MLT 1030/D, ADInstruments, PowerLab, Spain) under a tension of 0.5 g in a 25 ml chamber of an organ bath (ADInstruments, PowerLab, Spain). The temperature of the bathing buffer was 33 °C. Papillary muscles were equilibrated in the organ bath for 90 min. For threshold, the muscles were exposed to an electrical-field stimulation at 1 Hz and then at 20% higher than the threshold to record the contractile force [20]. The papillary muscle excitation and contraction forces in all four groups were recorded and analyzed.

### Evaluation of oxidative stress markers

Heart tissues were homogenized by a mechanical homogenizer and centrifuged at 15,000 g for 20 min. The supernatant was used for determination of the antioxidant levels in all treated groups by measuring the levels of reduced glutathione (GSH), lipid peroxidation, and SOD activity. Protein content was determined spectrophotometrically at 562 nm using a BCA Protein Assay Kit.

#### Determination of GSH

To measure GSH, 20 mM phosphate buffer and 0.04% DTNB (5,5-dithiobis (2 nitrobenzoic acid)) in 1% sodium citrate was added to the supernatant of heart tissue homogenate and the absorbance was read at 412 nm.

#### Determination of lipid peroxidation

Oxidative stress results in lipid peroxidation and formation of malondialdehyde (MDA). MDA was assessed in the supernatant of heart tissue homogenate using a commercial MDA assay kit (Zellbio GmbH, Ulm, Germany). All the procedure was performed according to the manufacturer’s instructions. MDA level was measured colorimetrically on an acidic medium at the temperature of 90–100 °C at 535 nm.

#### Determination of SOD activity

The total activity of SOD was measured in the supernatant of heart tissue homogenate by the Cayman Chemical SOD Assay kit (USA) which utilizes a tetrazolium salt to detect the generated superoxide radicals by measuring the absorbance at 420 nm.

### Histopathological evaluation

For histopathological assessments, the hearts were fixed in 10% formalin. Specimens of left ventricular free walls were embedded in paraffin blocks, cut into 5-μm thickness sections, stained with hematoxylin and eosin (H&E), and were analyzed (× 40; Olympus-2B microscope). Modified histopathological evidence were evaluated according to the procedure previously described [21].

### Statistical analysis

Data were expressed as the mean ± SEM. Statistical analysis was carried out by one-way ANOVA followed by Tukey’s multiple comparison test using GraphPad Prism 6.01 for Windows (GraphPad Software Inc., La Jolla, CA, USA). The *P* value was considered significant when *P* < 0.05.

## Results

### Effect of STS treatment on body weight of rats

**Fig. 2:**
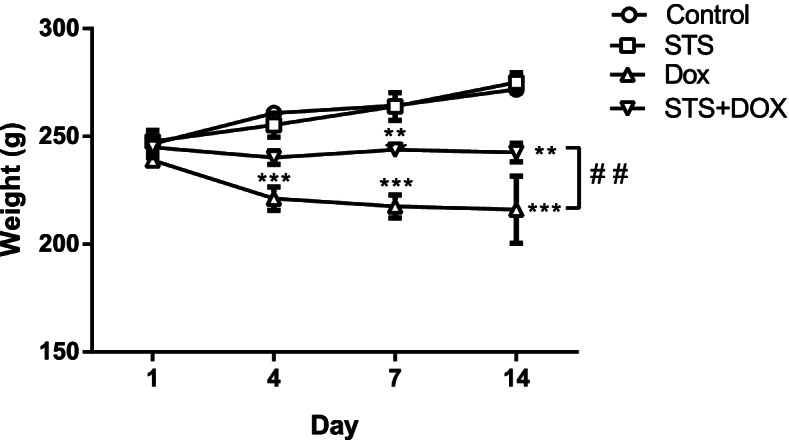
The body weight changes of rats in different groups during two-week study. Data were expressed as means±SEM of body weight in rats (*n* = 8). **P < 0.01, ***P < 0.001 compared with control group; ##*P* < 0.01 compared with DOX treated group

The changes of body weight of rats in different groups were monitored for 2 weeks (Fig. 2). The result showed that their weight in DOX-treated group was significantly lower than the control group (*P* < 0.001). However, the pre-treatment of DOX-treated rats with STS significantly improved their weight compared to DOX-treated group during the 2-week study (*P* < 0.01). There was no significant difference between STS and control groups. There was one death in DOX group and no deaths in the others groups.

### Protective effects of STS on DOX-induced ECG changes

**Fig. 3:**
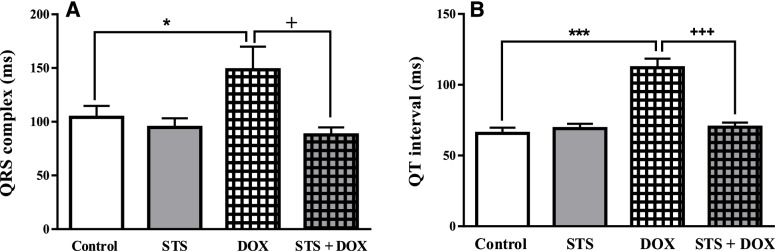
The effect of saline, STS (300 mg/Kg), DOX (2.5 mg/Kg), and STS+ DOX treatments on ECG parameters: QRS complex (**A**) and QT interval (**B**) in rats. Data were expressed as mean ± SEM (*n* = 8), one-way ANOVA followed by Tukey’s multiple comparison test. *P < 0.05, *** *P* < 0.001 as compared to the control group, + *P* < 0.05 and +++ *P* < 0.001 as compared to DOX group

The effects of pre-treatment with STS and/or DOX on ECG parameters were shown in Fig. 3A and B. The ECG images that were recorded at the end of the experiment (the last injection) were shown in supplementary Fig. 2. The results showed that DOX significantly induced an abnormally prolonged QRS complex and QT interval compared to the control group (*P* < 0.05 and *P* < 0.001, respectively), whereas pre-treatment of DOX-treated rats with STS could prevent this adverse effect of DOX on QRS complex and QT interval by significantly decreasing them (*P* < 0.05 and *P* < 0.001, respectively). No difference was seen in ST segment between treated groups compared to the control (Data not shown).

### Protective effects of STS on the papillary muscle excitation and contraction

**Fig. 4:**
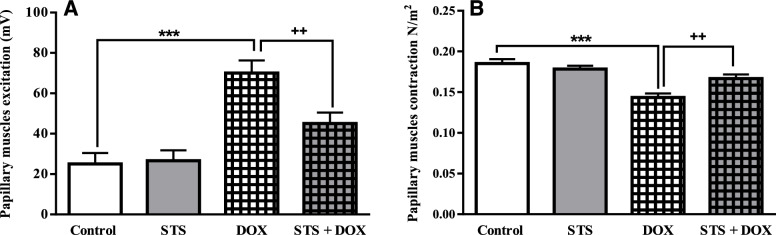
The effect of saline, STS (300 mg/Kg), DOX (2.5 mg/Kg), and STS+ DOX treatments on the papillary muscle excitation (**A**) and contraction (**B**) in rats. Data were expressed as mean ± SEM (*n* = 8). *** *P* < 0.001 compared with saline group, ++ *P* < 0.01 compared with DOX group

The effects of pre-treatment with STS and/or DOX on papillary muscle excitation and contraction were shown in Fig. 4A and B, respectively. The results showed that the papillary excitation threshold was significantly elevated in DOX-treated group compared to the control group (*P* < 0.001) and pre-treatment with STS significantly declined the threshold of excitation compared to the DOX-treated group (*P* < 0.01) (Fig. 4A). Also, DOX administration significantly reduced the contraction of the papillary muscle (*P* < 0.001), but pre-treatment with STS significantly prevented this adverse effect of DOX on the papillary muscle contraction by increasing it (*P* < 0.01) (Fig. 4B).

### The antioxidant effects of STS administration on DOX-treated rats

**Fig. 5:**
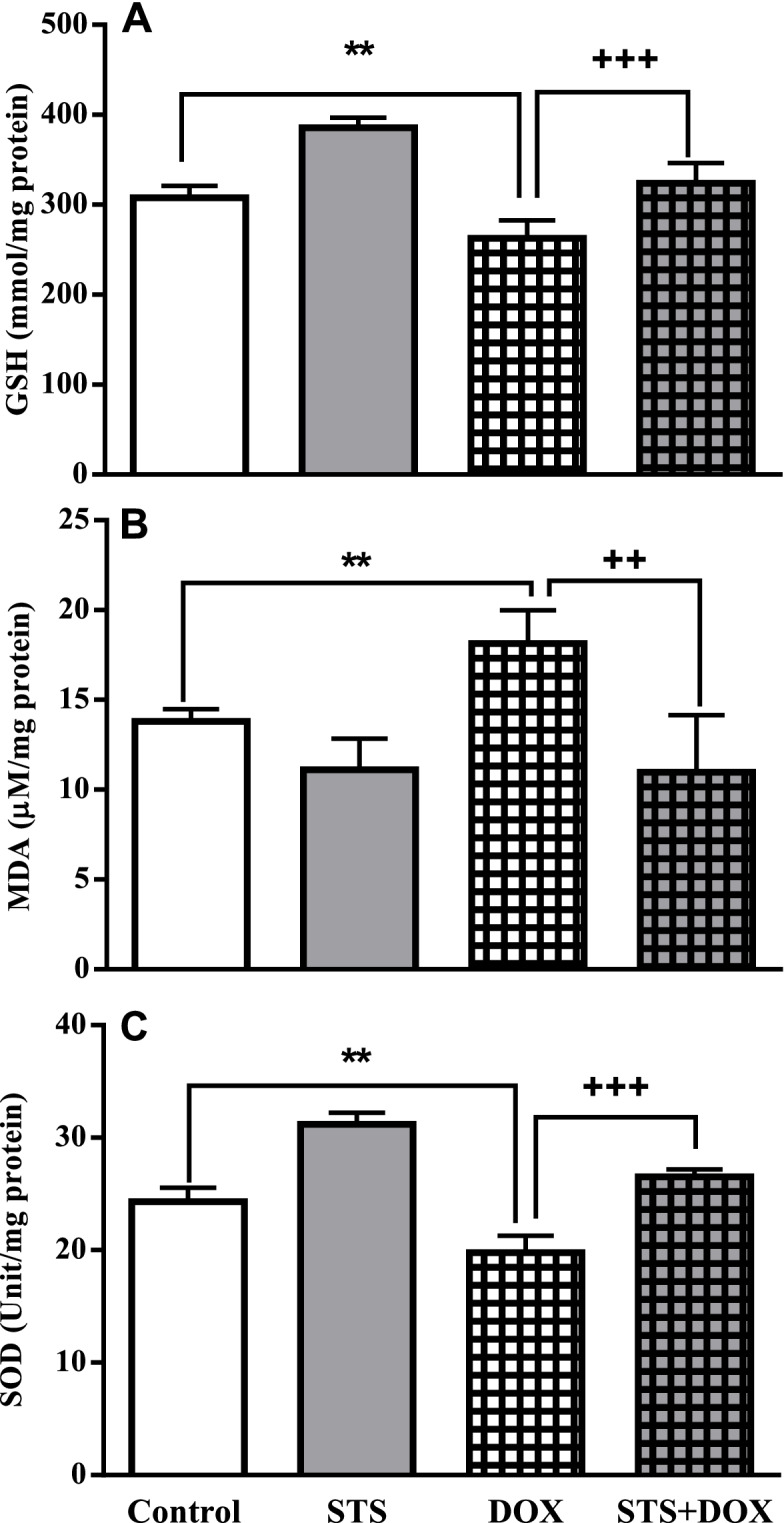
The effect of saline, STS (300 mg/Kg), DOX (2.5 mg/Kg), and STS+ DOX treatments on the antioxidant marker levels of GSH (**A**), lipid peroxidation (**B**) and SOD (**C**) in rats. Data were expressed as mean ± SEM (*n* = 8). ** *P* < 0.01 compared with saline group, ++ *P* < 0.01 and +++ *P* < 0.001 as compared to DOX group

The antioxidant levels in different treatment groups were evaluated by measuring GSH, MDA and SOD in heart tissues (Fig. 5A-C). The results revealed the increased oxidative stress level in heart tissues of DOX-treated rats by significant reduction in GSH content and SOD activity, as well as the elevated level of lipid peroxidation (MDA) compared with the control group (*P* < 0.01). The pre-treatment of DOX-treated rats with STS inhibited the oxidative stress process in heart tissues by significantly increasing the GSH content and SOD activity (*P* < 0.001), and decreasing the lipid peroxidation level (*P* < 0.01) compared to DOX-treated group which revealed the antioxidant property of STS against DOX-induced cardiotoxicity.

### Histopathological assessment

**Fig. 6:**
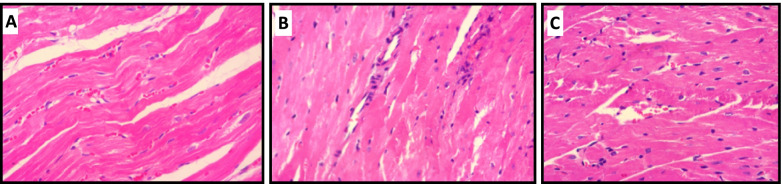
Representative photomicrographs of heart tissue sections with H&E staining after 2 weeks’ treatment with saline (**A**), DOX (2.5 mg/Kg) (**B**), and STS+ DOX (300 + 2.5 mg/Kg) (**C**). (**A**) The normal morphological appearance was observed in the control group, (**B**) DOX group rat showed mononuclear inflammation and cytoplasmic vacuolization, (**C**) STS + DOX group showed mild mononuclear inflammation and mild cytoplasmic vacuolization

The histopathological sections of rat cardiac tissues stained by H&E were shown in Fig. 6. The cardiac tissues injury were grading according to the 0–3 scale established by Billingham et al. in which the exhibition of cytoplasmic vacuolation and/or myofibrillar loss were considered as injuries [21] (Table 1). The image of control group showed no tissue disarrangement, hemorrhage, necrosis, and inflammatory cells with normal cardiomyocytes and vessels (Fig. 6A). DOX-treated group exhibited myocardial hypertrophy, mononuclear inflammation, myofibrillar loss, perinuclear and cytoplasmic vacuolization (Fig. 6B). Pre-treatment of DOX group with STS could significantly improve the DOX-induced myocardial damage which has been observed by mild infiltration of inflammatory mononuclear cells without any cytoplasmic vacuolization and neutrophil (PMN) infiltration (Fig. 6C). These results suggested that STS suggested the cardioprotective effects against DOX-induced cardiomyopathy in rats.

**Table 1:** Evaluation of myocardium tissue damage in all treated groups analyzed by Billingham scoring rules (0 = no damage, 1 = 1–15%, 2 = 16–35%, 3 = 35–100%)

Group	Total number of rats	Score			
		0	1	2	3
Control	5	5	0	0	0
STS	5	4	1	0	0
DOX ^a**^	7	0	2	2	3
STS + DOX ^b*^	5	2	3	0	0

Statistical analysis was performed using Mann-Whitney U test

^a^Significantly different compared to control group

^b^Significantly different compared to DOX group

* *P* < 0.05; ** *P* < 0.01

## Discussion

DOX is still one of the most frequently used chemotherapy drugs in clinic, although DOX-induced adverse effects especially the cardiotoxicity are limited its long-term application. Although the exact mechanisms of DOX-induced cardiotoxicity are not completely elucidated, the excessive intracellular ROS production in myocardium has been believed to be one of the main mechanisms of the cardiotoxicity. Therefore, any agents with antioxidant property could be considered to improve the cardiac dysfunctions and toxicity.

In this study, the cardioprotective effects of STS on a rat model of DOX-induced cardiotoxicity were investigated. The results suggested that STS pre-treatment attenuated DOX-induced oxidative stress and improved the cardiac dysfunctions as determined by ECG, cardiac oxidative stress level, and histological findings. The protective effects of STS pre-treatment could be due to increase in the antioxidant enzyme activities result in suppression of the oxidative stress damage of DOX in cardiomyocytes. These findings suggested that pre-treatment with STS in DOX- treated group could be a possible therapy against DOX-induced cardiactoxicity which was the goal of this study.

The experimental model that applied in this study, i.p. injection of DOX 6 times every other day at 2.5 mg/kg, is one of the most suitable models recently used to induce acute cardiotoxicity in rats [22]. Due to the fast apoptotic damage of DOX on rat’s cardiomyocytes shortly after the drug administration, the pre-treatment or quick treatment is critical to prevent the irreversible apoptotic death of myocardial cells [23]. Therefore, a pre-treatment strategy with STS 30 minutes prior to DOX injection was applied in this study. The results were in line with previous studies that 15 mg/kg cumulative dose of DOX induced signs of systemic and acute cardiac toxicity in rats including significant lower body weight compared to the control group during the study period (Fig. 2) [20, 24, 25]. Several reports have revealed the direct toxic effects of chemotherapy drugs, such as doxorubicin, on the gastrointestinal tract including the toxic effects on intestinal mucosa which appear as mucositis, leads to reduce food intake and weight loss [20, 26–28]. Pre-treatment of DOX group with STS could significantly increase the body weight compared to the DOX-treated group during the experimental period of 2 weeks (Fig. 2).

The cardiotoxicity of DOX can be assessed by the abnormal ECG changes in patients [29]. The most common ECG changes in cardiotoxicity induced by DOX are widening of QRS complex, prolongation of QT, ST segment, and R-R interval, bradycardia, T-wave flattening, and a significant decrease in R wave voltage which overall reflected the inhomogeneity of ventricular depolarization and repolarization, as well as prolonged action potential duration (APD) [30, 31]. Our results showed significant QRS complex widening and QT interval prolongation in DOX treated rats compared to the control group as the signs of ventricular arrhythmias (Fig. 3A and B). The pre-treatment of DOX- treated rats with STS could significantly inhibit the QRS complex and QT interval changes induced by DOX which showed the cardioprotective effect of STS on ventricular depolarization (QRS complex) and ventricular repolarization activity (QT interval), result in improving the cardiac output in DOX-treated rats.

The alternations in contractility force and electrical excitability of the cardiac muscle were assessed in papillary muscle. It has been reported that DOX depresses cardiac contractility by modifying calcium-mediated excitation–contraction coupling in both in vivo and in vitro studies [1, 32]. The results of present study demonstrated that DOX significantly increased the papillary muscles excitation and decreased the contraction compared to the control group (Fig. 4A and B) which are in line with previous studies [7, 28]. Also, the pre-treatment of DOX-treated rats with STS attenuated the negative effects of DOX on papillary muscles contractility and improved their functions.

The protective action of STS against DOX-induced cardiotoxicity was apparent in histopathological assessments. The results revealed significant myofibrillar loss and cell vacuolization in DOX-treated rats compared to the control group which was markedly attenuated with STS pre-treatment (Fig. 6 and Table 1). Quantitatively, the extent of heart tissue damage in STS + DOX group did not exceed more than 15%, and no DOX-specific tissue changes were evident in two of five rats in this group (score as 0). Thus, the result of histopathological evaluation was also suggested that STS could mitigate DOX-induced cardiotoxicity in DOX-treated rats.

Different mechanisms have been suggested to explain DOX-induced cardiotoxicity. Cardiac oxidative stress revealed by ROS production was considered as the main mechanism of cardiomyopathy of DOX. Elevated ROS levels can lead to oxidative damage to DNA, proteins, and lipids, as well as mitochondrial dysfunction, in which finally result in cell death [2, 4]. ROS interrupts the antioxidant defense mechanisms by the depletion of cardiac GSH content and SOD activity lead to accumulation of lipid peroxidation product, MDA, in heart tissues [33]. The results of the present study clearly indicated the increased level of oxidative stress in heart tissues of DOX-treated rats revealed by the significant increase in MDA level coupled with marked decreases in GSH content and SOD activity (Fig. 5A-C).

The protective effects of various compounds with potent antioxidant properties against the cardiac dysfunction induced by DOX have been reported in animal models through their neutralizing action on DOX-induced oxidative stress [20, 25, 28, 34, 35]. The present study revealed the protective effect of STS on DOX-induced oxidative stress by suppressing the elevated ROS level as monitored by significantly enhanced the activities of GSH and SOD, as well as reducing the content of MDA in heart tissues (Fig. 5A-C). This was in line with a recent report that showed the antioxidant effects of STS in doxorubicin-induced cardiotoxicity in mice by significant elevated activity of SOD [18]. Indeed, previous studies demonstrated that STS increased the level of GSH and SOD activity, and declined the level of MDA in an in vitro ischemia/reperfusion model in cardiomyocytes which resulted in a significant reduction in the oxidative damage [15].

According to the results of the present study, the protective effect of STS against DOX-induced cardiotoxicity was mainly attributed to the suppression of oxidative stress and suggested the free-radical scavenging capacity of STS. Moreover, STS improved the cardiac tissue antioxidant capacity (GSH, SOD) in DOX-treated rats. Nguyen et al. reported that the 2 weeks of oral treatment with STS improved the systolic function, left ventricular wall hypertrophy, cardiac fibrosis and systemic oxidative stress in a rat model of hypertension through its potent antioxidant activity, as well as its vasodilating property [13]. The findings of their report suggested that STS might be beneficial in cardiovascular disorders associated with a reduced left ventricular contractile function such as DOX-induced cardiotoxicity. Evidences from the earlier studies revealed that STS has calcium chelation effect, anti-inflammatory property, modulates mitochondrial potassium ATP channel which overall preserves the complex structure of mitochondria in heart and kidney tissues, and acts as a hydrogen sulfide donor [15, 36].

Several strategies have been made to mitigate and minimize the life threatening DOX-induced cardiotoxicity in cancer patients including close monitoring, early detection, administration of analogues of DOX (epirubicin, idarubicin, and mitoxantrone), and liposomal DOX [37]. The only FDA-approved cardioprotective agent against DOX-induced cardiotoxicity is dexrazoxane. It successfully used in children with various solid and hematologic cancers and could significantly decrease the cardiotoxicity induced by anthracycline [38, 39]. Also, the cardioprotective effect of dexrazoxane has been observed in elderly patients with acute myeloid leukemia and small cell lung cancer, but it is not approved to use in adults with hematologic malignancies. Although it is a cardioprotective agent, its protection is not complete and a close monitoring is required for signs of cardiotoxicity in patients [39]. Therefore, the use of a safe cardioprotective compound is still a promising therapeutic approach in prevention of the cardiotoxicity induced by cancer therapy drugs. STS is an officially approved safe medicine and an in vitro study showed that it did not attenuate the anticancer property of DOX [18].

## Conclusion

In conclusion, our study demonstrated that STS preserved the heart tissue functions in a DOX-induced cardiotoxicity model in rats. The results indicated that the protective effect was due to the anti-oxidative properties of STS. The outcomes of the present study may suggest STS as a possible pre-treatment agent in DOX treatment to protect hearts from cardiotoxic effects of DOX.

## Supplementary Information

**Additional file 1.**

## Data Availability

All data generated or analyzed during this study are included in this published article [and its supplementary information files].

## Abbreviations

DOX: Doxorubicin
CHF: Congestive heart failure
ROS: Reactive oxygen species
SOD: Superoxide dismutase
STS: Sodium thiosulfate
ECG: Electrocardiography
GSH: Glutathione
MDA: Malondialdehyde
H&E: Hematoxylin and eosin
