# Supplementary material for: Cardioprotective effects of sodium thiosulfate against doxorubicin-induced cardiotoxicity in male rats
Source: BMC Pharmacol Toxicol. 2022 May 25;23:32. doi: 10.1186/s40360-022-00569-3 (PMC9131624; doi:10.1186/s40360-022-00569-3)
Supplement: Supplementary file 1 — Additional file 1. [file 40360_2022_569_MOESM1_ESM.docx]

**Supplemantry File:**

**Supplementary Figure. 1.** The effect of different doses of STS (200, 300 and 400 mg/kg) 30 min prior to DOX injection (2.5 mg/Kg) on the papillary muscle contraction in rats. STS at the dose of 300 mg/kg could significantly reverse the effect of DOX on the contraction of the papillary muscle. No protective effect was observed at STS 200 mg/kg. Data were expressed as mean ± SEM (n = 8), one-way ANOVA followed by Tukey's multiple comparison test. *** *P* < 0.001 compared with control group, ++ *P* < 0.01 and +++ *P* <0.001 as compared to DOX group.

**
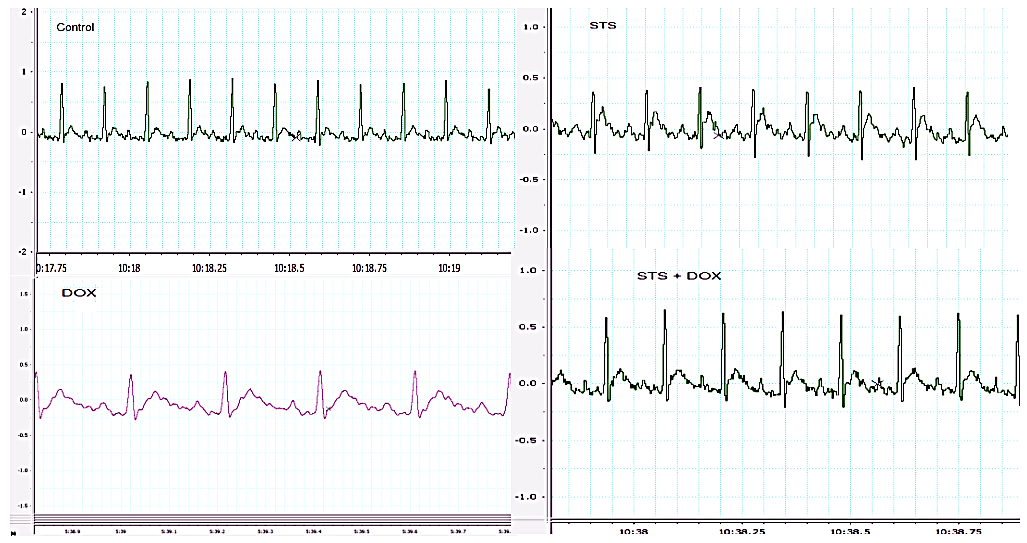
**

**Supplementary Figure. 2.** The effect of saline, STS (300 mg/Kg), DOX (2.5 mg/Kg), and STS+ DOX treatments on ECG patterns.
